# Intestinal Microbiota of Broiler Chickens As Affected by Litter Management Regimens

**DOI:** 10.3389/fmicb.2016.00593

**Published:** 2016-05-18

**Authors:** Lingling Wang, Mike Lilburn, Zhongtang Yu

**Affiliations:** ^1^Department of Animal Sciences, The Ohio State UniversityColumbus, OH, USA; ^2^Department of Animal Sciences, Ohio Agriculture Research and Development CenterWooster, OH, USA

**Keywords:** bacteria, broiler chickens, gastrointestinal microbiota, litter, poultry, pyrosequencing

## Abstract

Poultry litter is a mixture of bedding materials and enteric bacteria excreted by chickens, and it is typically reused for multiple growth cycles in commercial broiler production. Thus, bacteria can be transmitted from one growth cycle to the next via litter. However, it remains poorly understood how litter reuse affects development and composition of chicken gut microbiota. In this study, the effect of litter reuse on the microbiota in litter and in chicken gut was investigated using 2 litter management regimens: fresh vs. reused litter. Samples of ileal mucosa and cecal digesta were collected from young chicks (10 days of age) and mature birds (35 days of age). Based on analysis using DGGE and pyrosequencing of bacterial 16S rRNA gene amplicons, the microbiota of both the ileal mucosa and the cecal contents was affected by both litter management regimen and age of birds. *Faecalibacterium, Oscillospira, Butyricicoccus*, and one unclassified candidate genus closely related to *Ruminococcus* were most predominant in the cecal samples, while *Lactobacillus* was predominant in the ileal samples at both ages and in the cecal samples collected at day 10. At days 10 and 35, 8 and 3 genera, respectively, in the cecal luminal microbiota differed significantly in relative abundance between the 2 litter management regimens. Compared to the fresh litter, reused litter increased predominance of halotolerant/alkaliphilic bacteria and *Faecalibacterium prausnitzii*, a butyrate-producing gut bacterium. This study suggests that litter management regimens affect the chicken GI microbiota, which may impact the host nutritional status and intestinal health.

## Introduction

The gastrointestinal (GI) tract of chickens harbors a complex microbiota that plays an essential role in nutrient digestion and absorption, immune system development, and pathogen exclusion (Yeoman et al., 2012; Pan and Yu, 2014). Previous studies have demonstrated that diet (Jia et al., 2009; Hammons et al., 2010) and feed additives (Amerah et al., 2011; Danzeisen et al., 2011; Rodriguez et al., 2012) can impact the chicken GI microbiota with respect to diversity, composition, and structure. Understandably, most of the previous studies focused on how feed and feed additives affect the prevalence of enteric pathogens, such as *Salmonella* (Santos et al., 2008; Peinado et al., 2012), *Clostridium perfringens* (Si et al., 2009; Wei et al., 2013a), and *Campylobacter jejuni* (Chinivasagam et al., 2010; Ridley et al., 2011). However, prevalence of these pathogens and the risk of associated diseases can be lowered by a healthy GI microbiota through colonization resistance and competitive exclusion (Wagner, 2006; Callaway et al., 2008; Kerr et al., 2013). Some studies have also suggested that certain commensal bacteria can positively affect the efficiency of feed utilization by broiler chickens (Stanley et al., 2012, 2013). These studies have advanced our understanding on how diet, feed additives, and antimicrobial growth promoters (AGP) modulate the GI microbiota of chickens (Wise and Siragusa, 2007; Gong et al., 2008; Santos et al., 2008; Danzeisen et al., 2011). Similar to what is observed in mammals, the GI microbiota of chickens develops in the early stage of life (particularly within the first 2 weeks). When young chicks are delivered from the hatchery to a chicken house (typically at the age of 1–2 days), their initial GI microbiota is very simple containing a very small number of bacteria belonging to a few species (Fonseca et al., 2011; Cox et al., 2012; Hiett et al., 2013). After being placed in commercial chicken houses where litter serves as the bedding material, chicks are exposed to several sources of bacteria that can gain entry into the immature gut. These exogenous sources of bacteria include litter materials, feed, water, and ambient air. Because there is little colonization resistance in the young GI tract, many bacteria can readily colonize therein. As young chicks grow, their GI microbiota undergoes a series of temporal successions (van der Wielen et al., 2002; Lu et al., 2003a) and becomes increasingly diverse and complex (Wei et al., 2013b). Beginning from approximately day one, chicks begin pecking at and consuming litter materials, inoculating their young GI tract with bacteria present in the litter. Therefore, litter can have a significant effect on the development process of GI microbiota and its eventual composition and structure in chickens (Garrido et al., 2004; Torok et al., 2009).

Commercial broiler production involves fairly short growth cycles (about 6–7 weeks per growth cycle). Each growth cycle begins with placement of young chicks at 1 day of age at a high density (< 0.1 m^2^) in chicken houses with litter as bedding materials on the floor. Poultry litter is a mixture of bedding materials (e.g., pine shavings) and chicken excreta that contain chicken GI bacteria, undigested feed, uric acid, and other substances of host origin. Several studies have documented that poultry litter contains a complex and dynamic microbiota, composed primarily of GI and environmental bacteria depending on the litter management regimens (Lu et al., 2003b; Lovanh et al., 2007). The composition and structure of litter microbiota can be affected by the bedding materials used (Torok et al., 2009). In the US, broiler chicken litter (primarily pine shavings) is commonly reused for 6 or more consecutive growth cycles before a thorough cleanout to reduce the cost of fresh litter materials and disposal of reused litter (Coufal et al., 2006). Repeated use of poultry litter results in considerable changes in the chemical and microbiological conditions of the litter, and poor litter management can lead to increased litter moisture with concomitant increases in ammonia, pH, and increased density and diversity of microbes (primarily bacteria) (Omeira et al., 2006; Cressman et al., 2010). Changes in the litter microbiota brought about by repeated litter reuse can serve as a driving force that shapes the chicken GI microbiota (Cressman et al., 2010) because exposure of young chicks to different bacterial inocula can profoundly affect GI microbiota development (Yin et al., 2010). In addition, reused litter was shown to harbor less *Salmonella* (Roll et al., 2011) and *Clostridium perfringens* (Wei et al., 2013a) but to enable *Campylobacter jejuni* and *C*. *coli* to survive longer compared to fresh litter (Kassem et al., 2010). Moreover, 2 recent studies have shown that reused litter can affect the immune system of chickens (Lee et al., 2011; Shanmugasundaram et al., 2012), which suggests that litter conditions can also affect the GI microbiota of chickens indirectly through their immune system.

Numerous studies have examined the microbiota in the GI tract of chickens or in poultry litter. However, only one study examined the microbiota both in the GI tract and in the poultry litter with an focus on the interaction between the 2 microbiotas (Cressman et al., 2010). That study revealed that the litter microbiota and the GI microbiota affected each other in a reciprocal manner and fresh litter resulted in increased diversity and predominance of environmental bacteria in the GI microbiota of young chicks, while reused litter increased the bacteria of gut origin (Cressman et al., 2010). While that study pioneered a new area of research, only a limited number of bacteria were identified because it used sequencing of clone libraries of 16S rRNA gene amplicons. Therefore, the extent to which the microbiotas of litter and of GI tract of chickens affect each other remains to be determined. The objective of the present study was to further investigate the reciprocal effect of the litter and GI microbiotas of broiler chickens using pyrosequencing. The results could be useful in understanding the relationship between the litter and gut microbiotas of chickens as it relates to improving the health and well-being of chickens through litter management.

## Materials and methods

### Litter management

The experiment was conducted over 6 consecutive growing cycles with each cycle lasting 6 weeks. There was a 2-week down period between 2 consecutive cycles. Broiler chicks at 1 day of age were purchased from a commercial hatchery, and 50 chicks were randomly placed in each of 16 floor pens (4.73 m^2^ floor area per pen) with pine shavings as litter. This placement density, 0.095 m^2^ per chicks, is similar to that at commercial chicken houses. The pens were allocated into 1 of 2 management groups. For one group (8 pens), the litter was cleaned out after each growth cycle, and fresh pine shavings were placed prior to the arrival of new chicks for the next growth cycle (referred to as fresh litter, FL). For the reused litter (RL) group (8 pens), the litter was piled up in the center of each pen at the end of each growth cycle and stored for 10 days (without mixing or turning) before being redistributed within the same pen, as it is commonly practiced in broiler houses in the U.S. About 5 cm of fresh pine shavings were “top-dressed” or added on top of the used litter 2–3 days prior to the arrival of new chicks for the next growth cycle. Six growth cycles were performed for both the FL and the RL groups in parallel so that RL was generated and differential seasonal impact on FL and RL was avoided. All pens were physically separated by 24-inch-high plastic barriers to prevent litter contamination between pens. Within each pen, there was a 0.28 m^2^ concrete floor section without litter near the entry to the pen so that daily watering and feeding management could be performed without stepping onto the litter. Water was provided by bell-shaped poultry Plasson drinkers, and feed was provided by trough-type feeders. Disposable shoe covers were used before entering each pen to minimize potential cross contamination between pens. All the birds were fed the same commercial type of corn-soybean meal-based diet that met the nutrient levels recommended by NRC (NRC, 1994). The chicks/chickens were cared and handled following the animal use protocols approved specifically for this study by The Ohio State University Institutional Animal Care and Use Committee.

### Sample collection

Samples of Ileal mucosa and cecal digesta were collected from 4 randomly selected chickens from each pen at days 10 and 35 of the 6th growth cycle as described previously (Cressman et al., 2010). The 2 samples days were chosen to represent young and mature broiler chickens. Briefly, the ileum between the Meckles diverticulum and the ileocecal junction was removed. After the digesta was flushed out with sterile buffered saline, the ileal mucosa was scraped off using sterilized microscope slides. The cecal luminal content was squeezed out of the cecum. Both the ileal mucosa and the cecal luminal content were collected from each of the sampled birds. At the end of 6th growth cycle, 6 litter samples were also collected from each pen from the area around the drinkers and feeders and along the side of each pen. The litter samples from each pen were thoroughly mixed using a blender to reduce heterogeneity. All the samples were stored at −80°C until further analysis. The ileal mucosa was chosen because it is the interface between the host and the small intestinal bacteria.

### DNA extraction, PCR, and DGGE analysis

The samples of ileal mucosa and of cecal content from the 4 sampled chickens of each pen at each age (day 10 and day 35) were pooled based on the same wet weight. Metagenomic DNA was extracted from each pen-based composite GI sample and from each pen-based composite litter sample using the repeated bead beating and column purification method (Yu and Morrison, 2004b). The V3 region of 16S rRNA gene was PCR amplified using bacteria-specific primers (357F: CCT ACG GGA GGC AGC AG and 518R: ATT ACC GCG GCT GCT GG) with the forward primer having a 40 bp GC clamp attached to its 5′ end (Yu and Morrison, 2004a). The confirmed amplicons were subsequently analyzed using DGGE with a 40–60% denaturing gradient as described previously (Yu and Morrison, 2004a). The DGGE profiles were analyzed using BioNumerics (V.5.1; Applied Maths, Inc., Austin, TX). The DGGE banding patterns were transformed into a binary (presence and absence of bands) correlation cross-product matrix and then subjected to principle component analysis (PCA) using the PC-ORD software (V.5.0, MJM Software, Gleneden Beach, OR) as described previously (Cressman et al., 2010).

### Pyrosequencing and data analysis

Preparation of amplicon libraries and pyrosequencing were done at the Research and Testing Laboratories (Lubbock, TX) as described previously (Kim and Yu, 2014). Briefly, amplicon libraries of the V1–V3 region was prepared using the primers Gray28F (5′-GAGTTTGATCNTGGCTCAG-3′) and Gray-519R (5′-GTNTTACNGCGGCKGCTG-3′). The amplicons were sequenced when pyrosequencing was the only next-generation sequencing technology that could produce about 500 bp reads. The 8 pen-based composite samples within each litter management regimen were pooled (the same DNA quantity from each DNA sample) into one litter type-based composite sample for the same sample type (ileal mucosa, cecal content, and litter) to reduce cost, increase depth coverage, and obtain an “average” appraisal of the microbiota of each sampling location at each age. Each composite sample (it either represented ileal or cecal samples from 32 chickens or represented 48 litter samples), one unique barcode was added between the primers and the adaptors A and B that are required by the Roche 454 FLX Titanium system. The pyrosequencing data were processed using the Qiime pipeline (Caporaso et al., 2010) for denoising, removal of chimeric sequences, and quality checking as described previously (Kim and Yu, 2014), except more stringent criteria at the split_library.py step (--min_seq_length 200, --max_seq_length 600, --min_qual_score 25, maximum number of ambiguous bases 6, maximum length of homopolymer run 6, maximum number of primer mismatches 1, maximum number of errors in barcode 0, sliding window test of quality scores 50). The quality-checked sequences were aligned against the Greengenes core set Gg_13_5_99, and the sequences that failed to align with the reference sequence set were excluded from further analysis. The aligned sequences were grouped into operational taxonomic units (OTU) using *de novo* OTU picking at a distance of 0.04, which allows similar clustering of species-equivalent OTU based on the V1–V3 region as a distance 0.03 based on full-length of 16S rRNA genes (Kim et al., 2011). The same number of sequences was used for each sample type (8470 sequences for the ileal mucosa samples, 3078 sequences for the cecal content samples, and 4752 sequences for the litter samples) to avoid impact from different numbers of sequences from different samples. The OTUs were classified to species or a higher taxon using the RDP Naïve Bayesian Classifier (Wang et al., 2007) implemented in Qiime against the default taxonomy file (gg_13_5_otus/taxonomy/97_otu_taxonomy.txt) and the reference sequence file (gg_13_5_otus/rep_set/97_otus.fasta) at the default confidence level (80%). Pair-wise comparison between the 2 litter management regimens was performed using the Library Compare function at RDP with a significant level of *p* < 0.001. Library Compare was used because it estimates the likelihood that individual taxa differ in frequency between 2 libraries using a statistical test that can compare transcript levels in “digital Northern” analysis (Audic and Claverie, 1997). Good's estimate of coverage was calculated for each sample using Qiime.

The distributions of the OTUs obtained were visualized using the heatmap and clustering method implemented in the software GAP (http://gap.stat.sinica.edu.tw/Software/GAP/) as described previously (Li et al., 2014, 2015). Briefly, the abundance (number of sequences) of these OTUs was first log transformed for normalization. Pearson's correlation coefficients were calculated to examine the community similarity among the samples. Hierarchical clustering trees were generated using the rank-two ellipse seriation method (Chen, 2002; Wu et al., 2010) to grouping of the microbiotas.

### Data availability

The pyrosequencing data are available in the MG-RAST database (http://metagenomics.anl.gov/) under the project ID #9131.

## Results

### DGGE profiles of the microbiota

**Figure 1:**
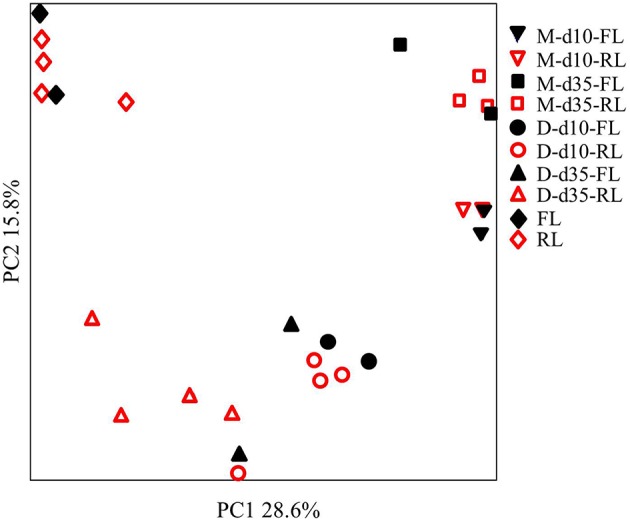
**Principal component analysis (PCA) plot of the DGGE profiling of the microbiome**. M, ileal mucosa; D, cecal digesta; d10 and d35, samples collected at 10 and 35 days, respectively, of bird age; FL, fresh litter; RL, reused litter. Some samples were superposed due to identical DGGE profiles.

All samples produced many DGGE bands (data not shown). PCA analysis of the DGGE profiles grouped the samples based on sampling types (litter vs. GI) or locations (ileal mucosa vs. cecal digesta), litter conditions (fresh vs. reused), and age (day 10 vs. day 35) (Figure 1). As shown with superposed sample symbols, some samples with each sample type (8 samples per sample type) had identical DGGE profiles. Small variance was explained by PC1 (28.6% of total variation) and PC2 (15.8%), reflecting relatively small differences in microbiota among the 8 pens of each sample type. For both the ileal mucosal and the cecal digesta samples, greater variability among replicate pens within each litter management regimen was noted at day 35 than at day 10.

### Alpha diversity of individual samples

In total, 147,670 quality-checked sequences were obtained and subjected to subsequent analysis. Estimate of Good's coverage reached >97.8% for all the samples, with higher coverage being achieved for the ileal mucosa samples (Table 1). The ileal mucosal bacterial community had much fewer species-equivalent OTUs, and a few of them dominated, resulting in low Shannon diversity index and evenness index. Compared to the cecal digesta samples, the litter samples had lower OTU richness. The phylum *Firmicutes* was dominant in all the samples at both ages. However, in the ileal mucosal samples, *Lactobacillales* was the most predominant order, while in the cecal digesta samples *Clostridiales* was most predominant (Supplementary Figures S1, S2). In the litter samples, the orders *Actinomycetales, Bacillales*, and *Lactobacillales* were more predominant than any other order (Supplementary Figure S3). More taxonomic orders of bacteria were also noted in the cecal and in the litter samples than in the ileal mucosal samples. More bacterial orders were identified in the fresh litter than in the reused litter.

**Table 1:** **Summary of alpha diversity of the GI microbiome and litter microbiome**.

**Sample type**	**Day**	**Litter condition+**	**Quality sequences^$^**	**Number of OTUs observed**	**Number of major OTUs observed^†^**	**Chao1**	**Shannon index**	**Evenness**	**Goods coverage**
Ileal mucosa	10	FL	10728	20	8	22	0.48	0.16	99.9%
		RL	8470	28	8	35	0.50	0.14	99.9%
	35	FL	12463	41	12	52	0.61	0.15	99.8%
		RL	9781	45	20	62	1.38	0.33	99.8%
Cecal lumen	10	FL	4803	221	92	236	3.91	0.72	98.2%
		RL	3078	282	124	371	4.51	0.76	97.4%
	35	FL	3631	317	146	390	4.68	0.78	97.0%
		RL	3218	315	141	382	4.60	0.77	97.2%
Litter^*^	35	fresh	4752	186	69	228	2.62	0.48	98.8%
		reused	4997	161	59	220	3.06	0.57	98.8%

+ , FL, fresh litter; RL, reused litter;

$ *Quality sequences indicate the sequences passing the quality control*.

* No liter sample was collected at day 10;

† *The OTUs representing ≥0.05% and ≥0.1% of total sequences in the ileal mucosa samples and the other samples, respectively*.

### Beta diversity among different samples

**Figure 2:**
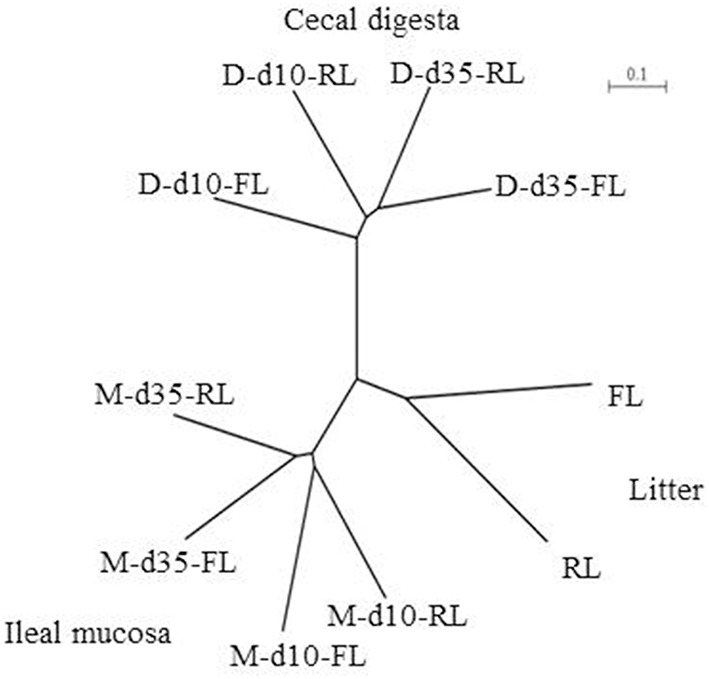
**Unweighted pair group method with arithmetic mean (UPGMA) tree based on a β-diversity distance matrix calculated using unweighted UniFrac metrics of the pyrosequencing data**. M, illeal mucosa; D, cecal digesta; d10 and d35, samples collected at 10 and 35 days, respectively, of bird age; FL, fresh litter; RL, reused litter.

The microbiotas were compared based on unweighted UniFrac analysis of the pyrosequencing data, and the comparison was visualized using a tree based on the unweighted pair group method with arithmetic mean (UPGMA). Similar to the comparison based on PCR-DGGE profiles, the pyrosequencing data also showed that the microbiotas were clustered in accordance with sample types, sampling locations, and the ages of birds (Figure 2). The fresh litter and the reused litter resulted in bifurcation of microbiota irrespective of sample type, sampling location, or age.

### Major taxa found in the samples

Numerous genera were found in the cecal digesta and the litter samples (Supplementary Table S1). Interestingly, Candidatus *Arthromitus*, a new proposed genus of segmented filamentous bacteria (Thompson et al., 2012), was the most predominant in the ileal mucosa at day 10 but gave way to *Lactobacillus* at day 35 (Table 2). The genera *Faecalibacterium, Butyricicoccus*, and an undefined candidate genus closely related to *Ruminococcus* (referred to *[Ruminococcus]* as used in the RDP database) were most prevalent in the cecal samples (Supplementary Table S1). The most predominant genus in both the fresh and the reused litters was *Corynebacterium*, while *Staphylococcus* was also predominant in the reused litter. Most members of these genera are either aerobic or facultatively anaerobic.

**Table 2:** **The most predominant genera in the ileal mucosa, cecal digesta, and litter samples**.

**Sample**	**Genus**	Relative abundance^*^			
		**Day 10**		**Day 35**	
		FL^**^	RL^**^	FL^**^	RL^**^
Ileal mucosa	*Candidatus Arthromitus*	++++++	++++++	+	+
	*Lactobacillus*	++	++	+++++++	++++++
Cecal content	*Bacteroides*	+	+	+	+
	*Butyricicoccus*	+	+	+	+
	*Faecalibacterium*	+	+	+	++
	*Lactobacillus*	++	+	+	+
	*Oscillospira*	+	+	+	+
	*Ruminococcus*	+	+	+	+
	[*Ruminococcus*]	++	+	++	+
Litter^***^	*Brachybacterium*			+	+
	*Brevibacterium*			+	+
	*Corynebacterium*			++++	+++
	*Facklamia*			+	+
	*Lactobacillus*			+	+
	*Sphingobacterium*			++	
	*Staphylococcus*			+	++

* *+, less than 10%; ++, 10–20%; +++, 20–30%; ++++, 40–50%; +++++, 70–80%; ++++++, 80–90%; +++++++, > 90%*.

** *FL, fresh litter; RL, reused litter*.

*** *No litter samples were collected at day 10*.

**Figure 3:**
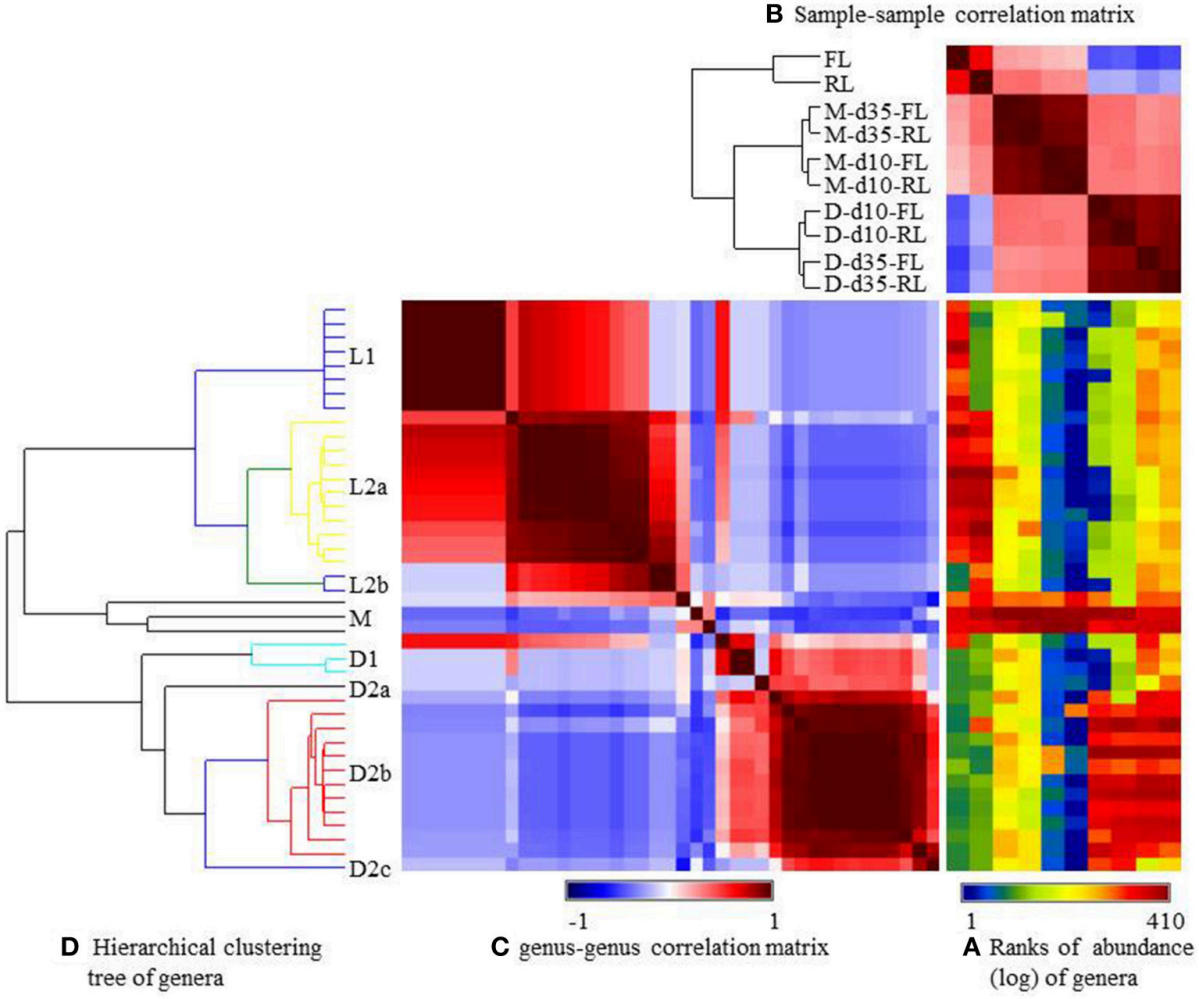
**Generalized association plots for the major genera identified in the 10 datasets. (A)** Genus abundance heatmap, **(B)** The sample-sample correlation map, **(C)** The genus-genus correlation map, and **(D)** The hierarchical clustering tree for sorting the genus-genus correlation map in **(C)**. The 10 datasets and the genera were sorted based on corresponding sample-sample correlation and genus-genus correlation, respectively. M, illeal mucosa; D, cecal digesta; d10 and d35, samples collected at 10 and 35 days, respectively, of bird age; FL, fresh litter; RL, reused litter.

The relative abundance of the major genera was visualized using heatmap (Figure 3) and their occurrence was listed in Supplementary Table S2 (the Genera spreadsheet). Consistent with the UniFrac analysis (Figure 2), the microbiotas were grouped, based on Pearson's coefficients (Figure 3B), primarily by sample types and to a lesser extent by litter management regimens. Based on distribution patterns, these genera were clustered into 5 groups (Figure 3D). Group L1 contained 8 genera with *Sphingobacterium* being most predominant, and they were found only in the fresh litter, while Group L2a (11 genera) was shared by both the fresh and the reused litter samples (Supplementary Table S2). Group L2b only had *Dialister* and *Oceanobacillus*, and it was only found in the reused litter. Overall, many genera found in the litter samples were not common or predominant GI bacteria. Group M contained *Enterococcus, Lactobacillus*, and Candidatus *Arthromitus*, and Candidatus *Arthromitus* was most predominant in the ileal mucosa at day 10. Group D1 contained 3 genera found only in the cecal digesta collected at day 35 from the chickens reared on the fresh litter (referred to as fresh-litter chickens). Group D2 contained 3 subgroups that were found almost exclusively in the cecal digesta samples. Group D2a (only 1 genus) was found in the cecal digesta collected at day 35 from the chickens reared on the reused litter (referred to as reused-litter chickens). Group D2b (12 genera) was shared by all the cecal digesta samples, while Group D2c (1 genus) was found in the cecal digesta samples of the fresh-litter chickens collected at day 10. All the genera found in the cecal digesta were common and/or predominant GI bacteria, such as *Ruminococcus, Clostridium, Blautia, Bacteroides*, and *Faecalibacterium*.

To identify the cecal bacterial genera that significantly differed between the 2 litter management regimens, the sequences representing known genera were compared between the 2 litter management regimens using the RDP Library Compare function, which can detect differentially represented taxa between 2 samples (Cole et al., 2009). Numerous genera differed in relative abundance between the 2 litter management regimens, including *Lactobacillus*, the *Escherichia*/*Shigella* group, *Bacteroides, Subdoligranulum*, and *Clostridium* XIVb for the litter samples, and *Blautia, Faecalibacterium*, and *Anaerotruncus* for the cecal digesta samples (Table 3). Overall, more cecal bacterial genera differed in relative abundance at day 10 than at day 35. *Faecalibacterium* and *Oscillibacter* were more predominant in cecum of the reused-litter chickens at day 35, while *Subdoligranulum* was more predominant in the fresh-litter chickens at day 10. The reused litter increased the relative abundance of *Enterococcus* but decreased that of *Lactobacillus* in the ileal mucosa samples. The 2 litter conditions also affected the relative abundance of some genera of bacteria in the litter samples, particularly *Corynebacterium, Facklamia, Escherichia/Shigella*, which were more predominant in the fresh litter, and *Yaniella, Staphylococcus, Brevibacterium, Salinicoccus*, which were more predominant in the reused litter.

**Table 3:** **Genera that differed in relative abundance (% of total sequences) between the fresh and the reused litter conditions**.

**Samples**	**Day**	**Genera**	**Fresh litter**	**Reused litter**	***P*-values**
Litter	35^*^	*Escherichia/Shigella*	1.2	0.0	3e-18
		*Brevibacterium*	0.3	3.6	6e-14
		*Corynebacterium*	49.1	26.6	6e-14
		*Facklamia*	7.3	1.9	6e-14
		*Lactobacillus*	1.9	5.6	6e-14
		*Staphylococcus*	2.1	21.4	6e-14
		*Yaniella*	0.5	3.1	6e-14
		*Salinicoccus*	0.3	1.6	1e-11
		*Brachybacterium*	4.7	7.6	4e-9
		*Acinetobacter*	0.6	0	4e-9
		*Oligella*	0.5	0	3e-8
		*Aerococcus*	1.7	3.1	3e-6
		*Paenalcaligenes*	0.3	0	1e-5
		*Dialister*	0	0.3	1e-5
		*Pseudomonas*	0.3	0	4e-5
		*Luteimonas*	0.3	0	2e-4
		*Alcaligenes*	0.2	0	4e-4
		*Atopostipes*	0.9	1.6	8e-4
Ileal mucosa	10	*Enterococcus*	0.07	0.76	6e-14
	35	*Lactobacillus*	96.2	90.3	6e-14
Cecal digesta	10	*Blautia*	0.2	2.1	6e-14
		*Escherichia*/*Shigella*	2.5	0.4	2e-12
		*Lactobacillus*	11.2	6.6	5e-12
		*Faecalibacterium*	4.7	8.4	8e-11
		*Bacteroides*	2.9	0.8	5e-10
		*Subdoligranulum*	4.9	2.4	7e-08
		*Anaerotruncus*	0.5	1.5	7e-06
		*Clostridium XlVb*	1.8	0.9	7e-04
	35	*Faecalibacterium*	5.1	17.0	6e-14
		*Oscillibacter*	0.2	1.5	5e-10
		*Subdoligranulum*	2.7	1.3	6e-05

* *No litter sample was collected at day 10*.

**Figure 4:**
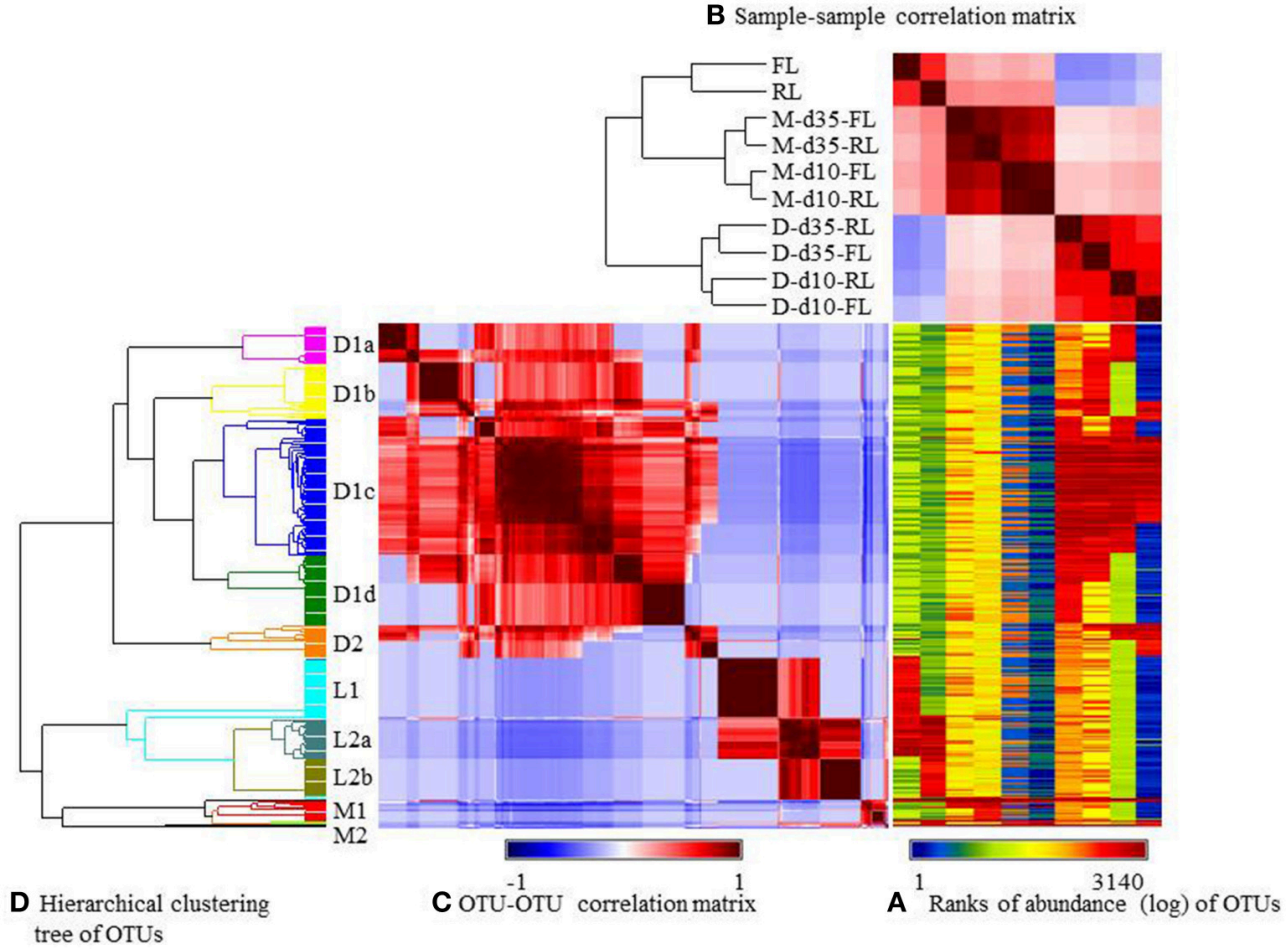
**Generalized association plots for the major OTUs identified in the 10 datasets. (A)** OUT abundance heatmap, **(B)** The sample-sample correlation map, **(C)** The OTU-OTU correlation map, and **(D)** The hierarchical clustering tree for sorting the OTU-OTU correlation map in **(C)**. The 10 datasets and the OTUs were sorted based on corresponding sample-sample correlation and OUT-OUT correlation, respectively. M, illeal mucosa; D, cecal digesta; d10 and d35, samples collected at 10 and 35 days, respectively, of bird age; FL, fresh litter; RL, reused litter.

The relative abundance of the major OTUs was visualized using heatmap (Figure 4) and their occurrence was listed in Supplementary Table S2 (the OTUs spreadsheet). Similar as at genus level, the microbiotas was mostly influenced by sample types and to a lesser extent by litter management regimens (Figure 3B). Three large groups of OTUs were found, each corresponding to a sample type: cecal digesta (designated as D), litter (designated as L), and ileal mucosa (designated as M) (Figure 4D). Group D1a contained 25 OTUs, including 17 being found only in the reused-litter chickens at day 10 and 8 also being found in the fresh-litter chickens at day 35 (Supplementary Table S2). Containing 34 OTUs, Group D1b was found only in the fresh-litter chickens at day 35, except 7 and 10 OTUs that were also found in the reused-litter chickens at day 35 and in the fresh-litter chickens at day 10, respectively. Group D1c contained the most number of OTUs (85 in total), and most of them were shared by all the cecal digesta samples. Consisting of 44 OTUs, Group D1d was found in the reused-litter chickens at day 35, with some OTUs being also found in the fresh-litter chickens at day 35. Group D2 contained 20 OTUs, which were found primarily in the fresh-litter chickens at day 10. The genus *Ruminococcus*, order *Clostridiales*, and family *Lachnospiraceae* were represented by the most numbers of OTUs of the cecal digesta.

The OTUs found in the litter samples had distinct distribution patterns, with Group L1 (37 OTUs) being exclusively found in the fresh litter, Group L2b (25 OTUs) exclusively in the reused litter, and Group L2a (26 OTUs) shared by both types of litter. The family *Sphingobacteriaceae* and the genera *Trichococcus, Acinetobacter, Corynebacterium, Leucobacter*, and *Facklamia* were the largest taxa represented by the Group L1 OTUs. The Group L2a OTUs were primarily assigned to the genera *Staphylococcus* and *Brachybacterium* and the family *Bacillaceae*, while those of Group L2b were mainly assigned to *Bacillaceae, Actinomycetales*, and *Corynebacterium*. Group M1 contained many OTUs assigned to *Lactobacillus*, while Group M2 contained only 2 OTUs assigned to *Enterococcus* and Candidatus *Arthromitus*. Three OTUs assigned to *Lactobacillus* and one OTU of Candidatus *Arthromitus* in these 2 OTU groups were found in all the samples, while the remaining OTUs were mostly found in the ileal mucosal samples.

**Figure 5:**
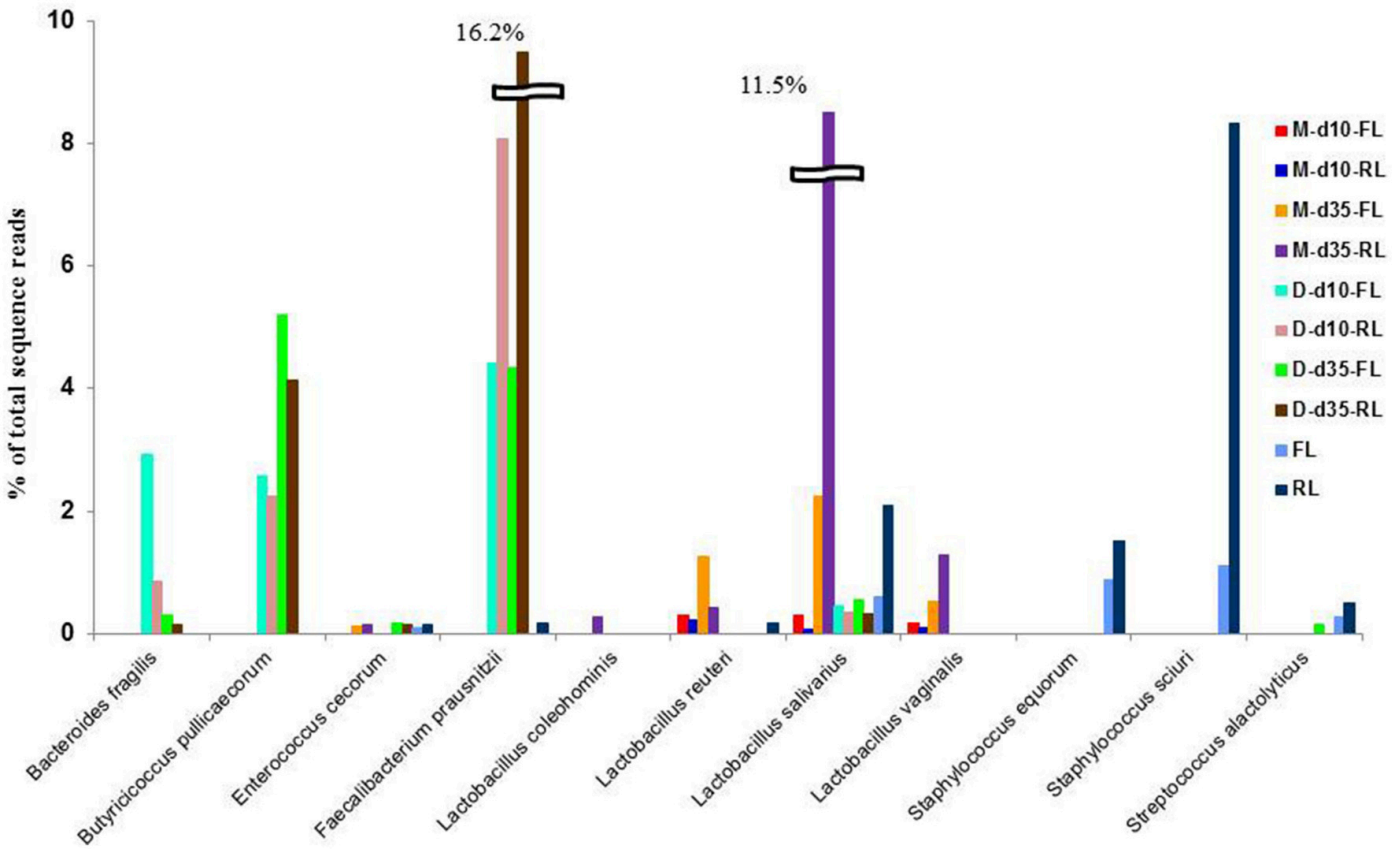
**Prevalence of some predominant bacterial species in the samples**. M, illeal mucosa; D, cecal digesta; d10 and d35, samples collected at 10 and 35 days, respectively, of bird age; FL, fresh littler; RL, reused litter.

The representative sequences of the 314 major OTUs (representing >0.05% and >0.1% of total sequences in at least one ileal mucosal sample and one cecal or litter sample, respectively) were classified using the Greengenes taxonomy database included in Qiime. In total, 133 OTUs were assigned to species within 41 genera, including 35 assigned to 19 known species within 15 genera. Amongst these species, 1, 4, 2, and 1 were assigned to *Bacteroides, Lactobacillus, Staphylococcus*, and *Faecalibacterium*, respectively. Most of these species, including *Bacteroides fragilis, Butyricicoccus pullicaecorum, F. prausnitzii, L. salivarius, L. vaginalis, Staphylococcus equorum*, and *S. sciun*, differed significantly in relative abundance between the 2 litter management regimens (Figure 5). Of the identified species, *L. salivarius* is the most dominant in the ileal mucosa samples, which increased from about 0.1% of total sequence reads at day 10 to >10% at day 35, suggesting temporal shift of mucosal bacteria as chickens grew. Although, the order *Clostridiales* was dominant in the cecal digesta samples (Supplementary Figure S2), no known species was predominant except *F. prausnitzii* and *B. pullicaecorum*, indicating greater diversity of unknown species in the cecal digesta than in the ileal mucosa.

## Discussion

Understanding the reciprocal impact between litter microbiota and chicken GI microbiota is important to guide proper management of poultry litter and bird health, especially as antibiotic growth promoters will be phased out in the US. This study for the first time used 16S rRNA gene-based metagenomic analysis in examining such reciprocal impact in a comprehensively manner. Consistent with a previous study (Cressman et al., 2010), fresh litter and reused litter differed in microbiota, but the present study revealed much greater bacterial diversity (21 vs. 2 genera) and more detailed differences in microbiota composition between the two litter management regimens. Five of the 8 major genera that were found only in the fresh litter (Group L1 genus) belong to *Proteobacteria*. Although, the predominance of *Proteobacteria* and occurrence of *Pseudomonas* (not a major genus) was also reported in the fresh litter in the previous study (Cressman et al., 2010), the present study documented a greater diversity of environmental bacteria in the fresh litter. Interestingly, *Oceanobacillus* and *Dialister*, which are alkaliphilic (Heyrman and Vos, 2015) and bile-tolerant (Wade, 2015), respectively, were found only in the reused litter, and *Salinicoccus*, another halophilic genus (Ventosa, 2015), was found more predominant (near by 1 log) in the reused than in the fresh litter. These results suggest that the reused litter might have increased salt content due to repeated reuse and composting. Although the litter samples were not chemically analyzed and salt content in poultry litter has not been reported in the literature, salt can accumulate in reused litter, especially when reused litter is composted as done in the present study. It should be noted, the litter in the previous study was sampled after 2 years, while in the present study the litter was sampled after about 1 year reuse. The length of litter reuse might probably have affected the effect of litter management regimens.

Achieving coverage >97%, this study identified nearly all the bacteria present in the samples. A close examination of the lineage of the major OTUs showed that although some genera were shared between the fresh and the reused litter, some OTUs were found only in one of the two litter types. These results suggest impact of the litter management regimens on litter microbiota at species level. The fresh litter contained bacteria that are not commonly found in GI tract but in other environments, such as the OTUs (Group L1 OTUs) assigned to *Acinetobacter, Devosia, Luteimonas, Trichococcus*, and *Yaniella*. These bacteria were probably derived from the fresh litter material or acquired during the growth cycles, and they were not competitive in the reused litter. On the other hand, some OTUs (Group L2b OTUs) were only found in the reused liter and they are members of halo- or bile-tolerant genera (e.g., *Salinicoccus, Oceanobacillus*, and *Dialister*). These OTUs might represent bacteria adapted to the reused litter. Among the OTUs (Group L2a) that were shared between the two types of litter, some represent common genera found in GI tract, such as *Enterococcus, Streptococcus, Facklamia*, and *Brachybacterium*, while others represent genera that were also found in fresh litter (Group L1 OTUs). These latter bacteria might be from the initial litter material and/or the environment but had become adapted to the reused litter conditions. Nevertheless, *Salmonella, Clostridium perfringens*, or *Campylobacter jejuni* was not detected in the fresh or the reused litter. This concurs with the similar mortality rates between the 2 litter management regimens (data not shown). Future studies using qPCR are needed to determine if litter management regimens can significantly affect the prevalence and abundance of these enteric pathogens.

This study is congruent with several previous studies with respect to revelation of the major groups of GI bacteria, such as *Lactobacillus* in ileal mucosa and *Clostridia* in cecal lumen (Gong et al., 2002, 2007; Lu et al., 2003b; Choi et al., 2014). The predominance of *Facklamia, Salinicoccus*, and *Corynebacterium* also corroborates the finding of a previous study (Lu et al., 2003a). However, this study revealed impact of litter management (fresh vs. reused) on the microbiota in both ileal mucosa and cecal digesta. First, no genus was only found in the ileal mucosa corresponding to either litter type. However, the litter management regimens affected the predominance of *Enterococcus* and *Lactobacillus*, with the reused litter favoring *Enterococcus* in the ileal mucosa at day 10 but *Lactobacillus* at day 35 (Table 3), suggesting that reused litter may serve as a source of *Enterococcus* for young chicks. Second, *Lactobacillus* and Candidatus *Arthromitus* showed opposite temporal trends in predominance between the 2 ages irrespective of the litter management regimens, with the former being more predominant at day 35, while the latter more predominant at day 10. Candidatus *Arthromitus* was first proposed by Snel et al. (1995) to include segmented filamentous bacteria that have been reported in trout, mice, and rats. It has been reported 3 times in chickens, once in cecal content (Snel et al., 1995) and twice in mucosa (Gong et al., 2007). The Candidatus *Arthromitus* sequences we found and those identified in the gut of chicken, turkey, and rat (Snel et al., 1995; Gong et al., 2007; Danzeisen et al., 2013) are very similar. Having close contact with gut epithelial wall (Thompson et al., 2012), Candidatus *Arthromitus* was thought to modulate host immune response (Bolotin et al., 2014), but there is also a conflicting report (Thompson et al., 2013). Future studies are warranted to verify this dynamic trend and the potential biological importance to host.

No litter-specific genus was found, suggesting minor impact of the litter management on the bacteria present in ileal mucosa at genus level. However, OTUs specific to age or litter types were found, including 4 OTUs only found at day 35 and 7 OTUs found only at 35 in reused-liter chickens, and most of these OTUs belong to *Lactobacillus* (Supplementary Table S2). It is also of interest to note that the reused litter corresponded to more OTUs in the ileal mucosa than the fresh litter at day 35. These results suggest that litter management regimens can affect some of the ileal mucosal bacteria residing in an age-dependent manner. The association between *Lactobacillus* and chicken performance has been mixed. In one study, one bacterial OTU distantly related to *L*. *crispatus* was negatively associated with feed conversion efficiency (Stanley et al., 2012), but in another study, 2 OTUs related to *L. coleohominis* were positively associated with feed conversion efficiency (Stanley et al., 2013). In a third study (Torok et al., 2011), bacterial phylotypes related to *L. salivarius, L. aviarius*, and *L. crispatus* were found to be associated with decreased bird performance. Because litter management can affects occurrence of *Lactobacillus*, the indirect effect of litter management through effect on *Lactobacillus* warrants further research.

In the cecal digesta samples, the reused litter resulted in greater species richness than the fresh litter at day 10 but not at day 35. In addition, more genera in the cecal digesta differed in relative abundance at day 10 than at day 35 (Table 3, Figure 3). These results suggest that litter management regimens can have more profound impact to the GI microbiota of young chicks than to that of mature birds. The decrease in the litter effect with age might best be explained by the increasing species richness, and thus colonization resistance (Spees et al., 2013), in mature birds. It may also be a reflection of the accumulation of fecal bacteria in the fresh litter at day 35. *Blautia, Faecalibacterium*, and *Anaerotruncus*, all of which are common fecal bacteria, were more predominant in the cecal digesta of the reused-litter young chickens, suggesting that reused litter may expedite bacterial colonization of GI tract, and thus colonization resistance, in young chicks. On the other hand, the fresh litter resulted in more *Escherichia*/*Shigella, Lactobacillus, Bacteroides*, and *Subdoligranulum* in the cecal digesta (Table 3). These genera were probably less competitive in reused litter than in fresh litter. The previous study also noted decreased litter effect on the microbiota of cecal digesta than that of the ileal mucosa (Cressman et al., 2010). Given that the ileum is upstream of the cecum and that the ileum has lower microbial diversity than the cecum, this reduction in the litter effect along the GI tract is expected.

Greater litter effect on the cecal bacterial community was noted at species-equivalent OTU level than at genus level. Most of these OTUs were assigned to *Ruminococcaceae, Lachnospiraceae*, and *Clostridiales*. Although some of these OTUs were shared between the 2 litter management regimens (e.g., most of the Group D1c OTUs), many of them were found only at one of the 2 ages or corresponded to only one of the 2 litter types (Figure 3, Supplementary Table S2). Because most of the OTUs were not assigned to known species genera, their differential occurrence with respect to either age or litter type cannot be explained.

Butyrate is anti-inflammatory (Van Immerseel et al., 2010; Celasco et al., 2014), and some of the identified cecal bacteria were assigned to known butyrate-producing genera, such as *Faecalibacterium, Subdoligranulum*, and *Butyricicoccus* (Louis and Flint, 2009). *Faecalibacterium* was more predominant in the cecal digesta of reused-litter chickens, while *Subdoligranulum* showed the opposite trend at both ages (Table 3). A recent study showed that *Faecalibacterium* and *Subdoligranulum* constituted a single major group of cecal bacteria in conventionally reared chickens (Lund et al., 2010). *F. prausnitzii* has been reported to be anti-inflammatory in humans (Sokol et al., 2008), but it is not known if members of *Subdoligranulum* are anti-inflammatory. Because reused litter can induce inflammatory response in the intestine of chickens (Shanmugasundaram et al., 2012), future research is needed to determine if these butyrate producers contribute to the immune response induced by reared litter.

Collectively, halotolerant/alkaliphilic bacteria tended to increase in reused litter. Ileal mucosal bacterial community was affected more profoundly than cecal luminal bacterial community. Litter management regimens also had greater impact on gut bacterial community in young chicks than in mature birds. Some butyrate-producing bacteria and *Lactobacillus* were affected by litter management regimens, potentially affecting host health and feed conversion efficiency. Concurrent chemical analysis of litter material and bird performance is needed in future studies to determine how litter management affects bird health and performance.

## Author contributions

ML, ZY conceived the study and designed the experiments. LW did the data analysis and wrote the paper. ZY, ML revised the paper.

### Conflict of interest statement

The authors declare that the research was conducted in the absence of any commercial or financial relationships that could be construed as a potential conflict of interest.
