# Supplementary material for: Intestinal Microbiota of Broiler Chickens As Affected by Litter Management Regimens
Source: Front Microbiol. 2016 May 18;7:593. doi: 10.3389/fmicb.2016.00593 (PMC4870231; doi:10.3389/fmicb.2016.00593)
Supplement: Supplementary file 1 [file Table1.PDF]

Supplementary Table S1. Relative abundance (% of total sequences) of the known genera detected in all the samples collected in growth cycle 6†.

Taxon	Ileal Mucosa				Cecal Digesta				Litter	
	Day 10		Day 35		Day 10		Day 35		FL	RL
	FL*	RL*	FL	RL	FL	RL	FL	RL		
**p_*Actinobacteria***										
Brevibacterium									3.98	4.58
Corynebacterium			0.07						48.80	26.18
Brachybacterium									4.61	7.54
Dietzia									0.44	0.26
Leucobacter									0.17	
Yaniella									0.34	2.66
**p_*Bacteroidetes***										
Bacteroides					2.91	0.84	0.30	0.16		
Sphingobacterium									10.67	
**p_*Firmicutes***										
Oceanobacillus										0.10
Jeotgalicoccus									1.41	0.78
Salinicoccus									0.21	1.36
Staphylococcus				0.10					2.31	19.81
Aerococcus									1.66	2.76
Facklamia									7.55	1.70
Trichococcus									0.51	
Enterococcus	0.07	0.73	0.12	0.13	0.10		0.17	0.16	0.11	0.40
Lactobacillus	12.34	11.56	96.13	89.83	10.99	6.43	1.82	1.83	1.81	5.34
Streptococcus							0.14		0.27	0.50
Candidatus Arthromitus	87.29	86.87	3.24	8.80	0.50	1.88	2.48	3.29	0.82	1.40
Clostridium					0.19		0.80	0.40		
[Ruminococcus]					12.87	9.91	11.04	8.58		
Anaerovorax							0.11			
Blautia					0.17	2.01	2.40	1.96		
Coprococcus		0.07			0.37	0.26	0.55	0.59		
Dorea					0.48	0.62	0.83	0.68		
Anaerotruncus					0.21	0.36				
Butyricicoccus					2.56	2.24	5.21	4.13		
Faecalibacterium					4.41	8.09	4.35	16.22		0.18
Oscillospira					3.91	4.55	6.58	6.00		
Ruminococcus					1.35	2.83	3.33	3.39		
Dialister										0.34
[Eubacterium]					0.23	0.16	0.28	0.19		
cc_115							0.17			
Coprobacillus					0.44	0.26	0.30	0.28		
**p_*Proteobacteria***										
Devosia									0.13	
Alcaligenes									0.23	
Oligella									0.51	
Acinetobacter									0.67	
Pseudomonas							0.39		0.29	
Luteimonas									1.96	
**p_*Verrucomicrobia***										
Akkermansia								0.16		

†, the % values do not add up to 100 because the % of unclassified genera was not included; * FL, fresh litter, RL, reused litter
