# Supplementary material for: Intestinal Microbiota of Broiler Chickens As Affected by Litter Management Regimens
Source: Front Microbiol. 2016 May 18;7:593. doi: 10.3389/fmicb.2016.00593 (PMC4870231; doi:10.3389/fmicb.2016.00593)
Supplement: Supplementary file 3 [file Presentation1.PDF]

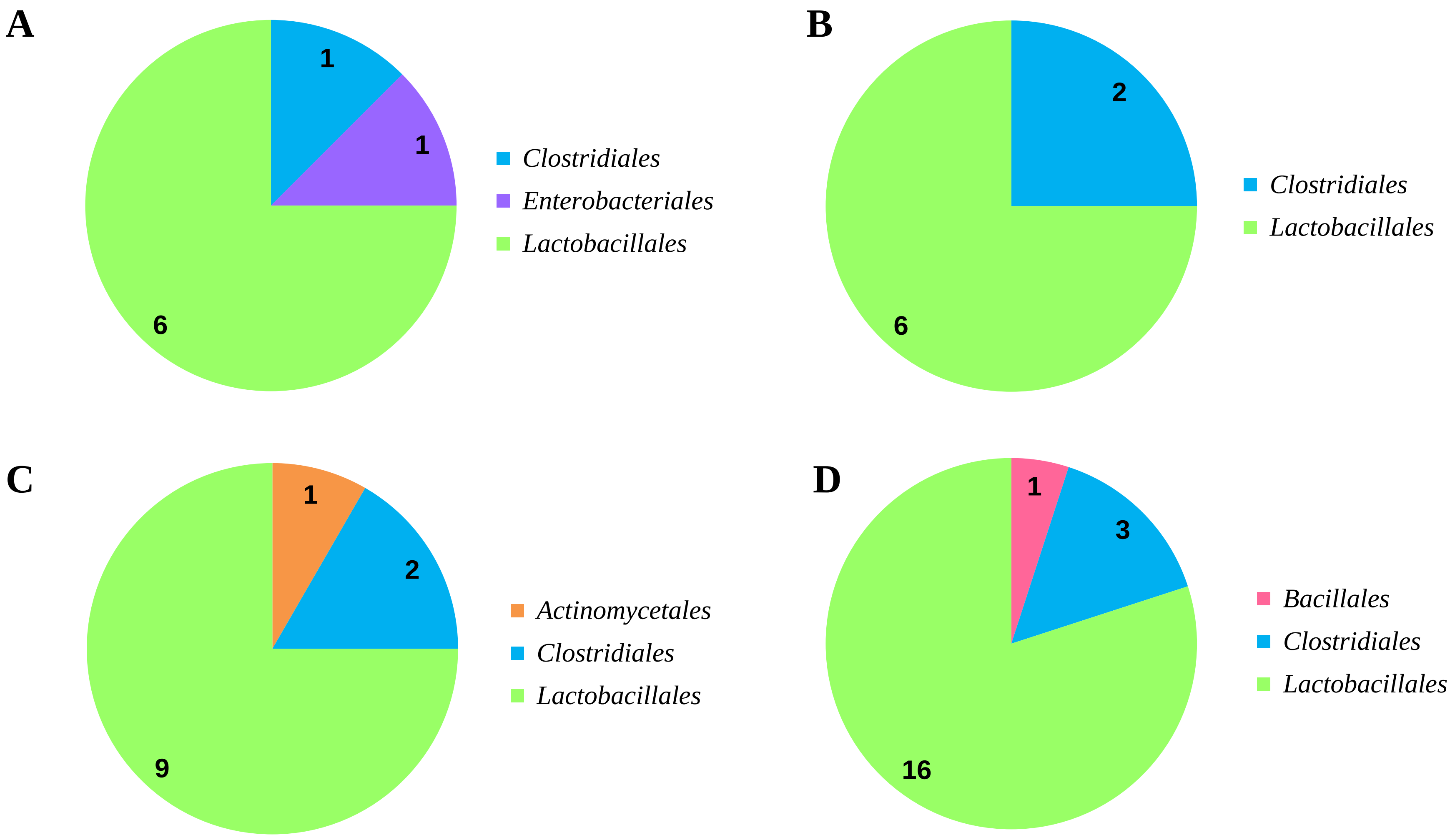

Supplementary Figure S1. Distributions of bacterial orders found in the ileal mucosal samples collected in growth cycle 6. A, fresh-litter chickens at day 10; B, reused-litter chickens at day 10; C, fresh-litter at day 35; D, reused-litter chickens at day 35.

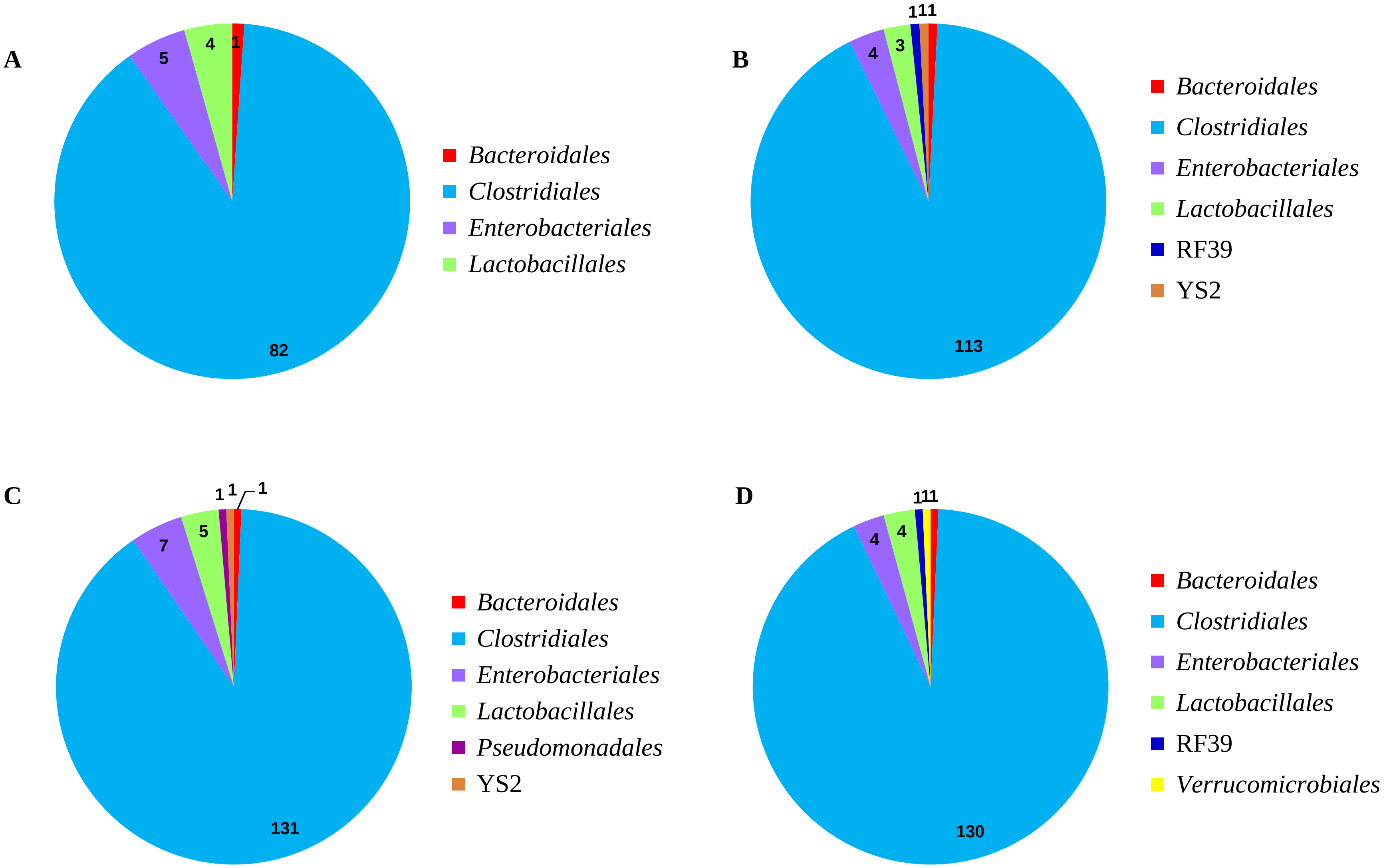

Supplementary Figure S2. Distributions of bacterial orders found in the ileal mucosal samples collected in growth cycle 6. A, fresh-litter chickens at day 10; B, reused-litter chickens at day 10; C, fresh-litter at day 35; D, reused-litter chickens at day 35.

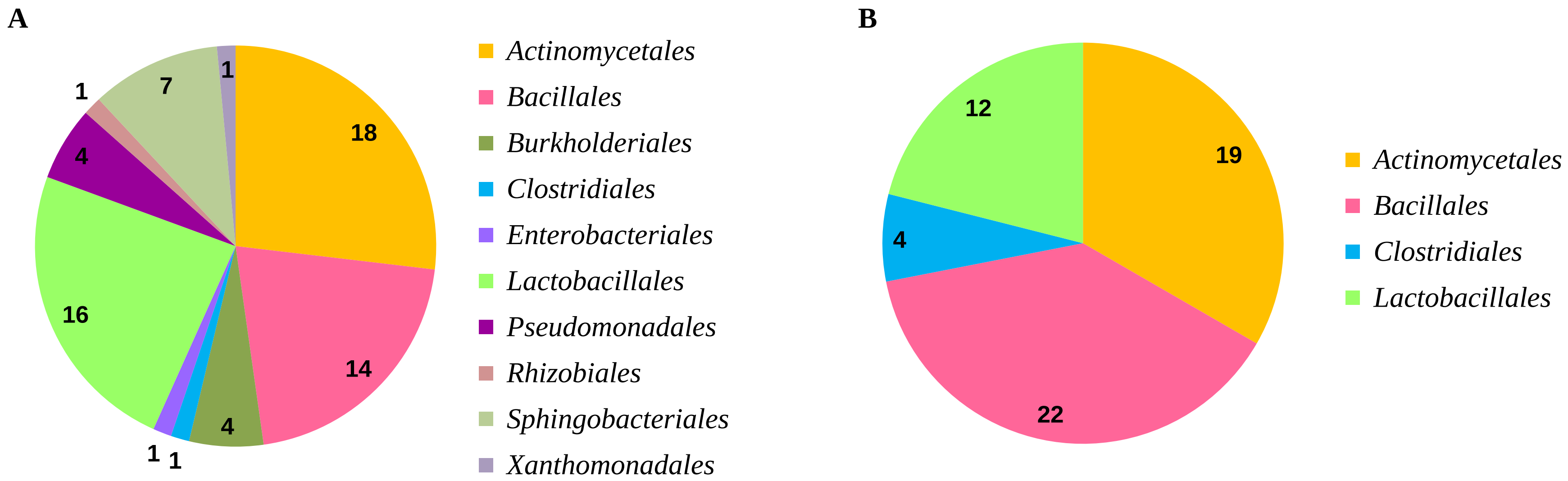

Supplementary Figure S3. Distributions of bacterial orders found in the fresh (A) and reused (B) litter in growth cycle 6.
